# Methyl 6-methoxy­carbonyl­methyl-2-oxo-4-phenyl-1,2,3,4-tetra­hydro­pyrimidine-5-carboxyl­ate

**DOI:** 10.1107/S1600536808026135

**Published:** 2008-08-16

**Authors:** Viktor Kettmann, Jan Světlík, Lucia Veizerová

**Affiliations:** a Faculty of Pharmacy, Comenius University, Odbojarov 10, SK-83232 Bratislava, Slovakia

## Abstract

The title compound, C_15_H_16_N_2_O_5_, belongs to the class of monastrol-type anti-­cancer agents and was selected for crystal structure determination in order to determine the conformational details needed for subsequent structure–activity relationship studies. The central tetra­hydro­pyrimidine ring has a flat-envelope conformation. The 4-phenyl group occupies a pseudo-axial position and is inclined at an angle of *ca* 90° to the mean plane of the heterocyclic ring. Of the two methyl ester groups, one (in the 5-position) is in a coplanar and the other (in the 6-position) in a perpendicular orientation with respect to the heterocyclic plane. The coplanar 5-ester group has its carbonyl bond oriented *cis* with respect to the pyrimidine C=C double bond. By comparison of the structural results for the present compound with those determined previously for its diethyl analogue, we have identified the mol­ecular factors which control the dual course of the Biginelli reaction with salicylaldehyde. The crystal structure is dominated by two hydrogen bonds which link the mol­ecules into chains of dimers.

## Related literature

For related literature, see: Haggarty *et al.* (2000 ▶); Hirshfeld (1976 ▶); Kettmann *et al.* (2008 ▶); Klein *et al.* (2007 ▶); Mayer *et al.* (1999 ▶); Světlík *et al.* (2008 ▶).
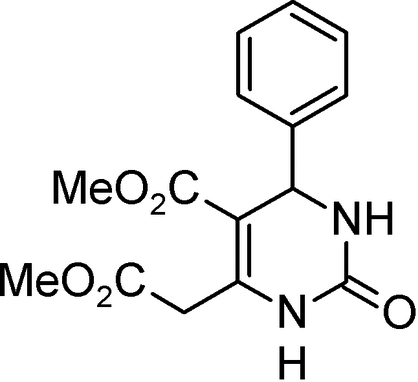


## Experimental

### 

#### Crystal data

C_15_H_16_N_2_O_5_
*M* *_r_* = 304.35
Monoclinic, 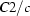

*a* = 23.498 (5) Å
*b* = 12.072 (2) Å
*c* = 10.933 (5) Å
β = 99.15 (2)°
*V* = 3061.9 (16) Å^3^
*Z* = 8
Mo *K*α radiation
μ = 0.10 mm^−1^
*T* = 296 (2) K
0.30 × 0.25 × 0.20 mm

#### Data collection

Siemens P4 diffractometer
Absorption correction: none
5187 measured reflections
4466 independent reflections
2308 reflections with *I* > 2σ(*I*)
*R*_int_ = 0.040
3 standard reflections every 97 reflections intensity decay: none

#### Refinement

*R*[*F* ^2^ > 2σ(*F* ^2^)] = 0.046
*wR*(*F* ^2^) = 0.118
*S* = 0.90
4466 reflections
201 parameters
54 restraints
H-atom parameters constrained
Δρ_max_ = 0.13 e Å^−3^
Δρ_min_ = −0.27 e Å^−3^

### 

Data collection: *XSCANS* (Siemens, 1991 ▶); cell refinement: *XSCANS*; data reduction: *XSCANS*; program(s) used to solve structure: *SHELXS97* (Sheldrick, 2008 ▶); program(s) used to refine structure: *SHELXL97* (Sheldrick, 2008 ▶); molecular graphics: *PLATON* (Spek, 2003 ▶); software used to prepare material for publication: *SHELXL97*.

## Supplementary Material

Crystal structure: contains datablocks global, I. DOI: 10.1107/S1600536808026135/bv2102sup1.cif

Structure factors: contains datablocks I. DOI: 10.1107/S1600536808026135/bv2102Isup2.hkl

Additional supplementary materials:  crystallographic information; 3D view; checkCIF report

## Figures and Tables

**Table 1:** Hydrogen-bond geometry (Å, °)

*D*—H⋯*A*	*D*—H	H⋯*A*	*D*⋯*A*	*D*—H⋯*A*
N1—H1⋯O1^i^	0.86	2.06	2.8326 (17)	149
N3—H3⋯O4^ii^	0.86	2.32	3.0730 (17)	146

Symmetry codes: (i) 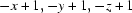; (ii) 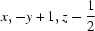.

**Fig. 1.**
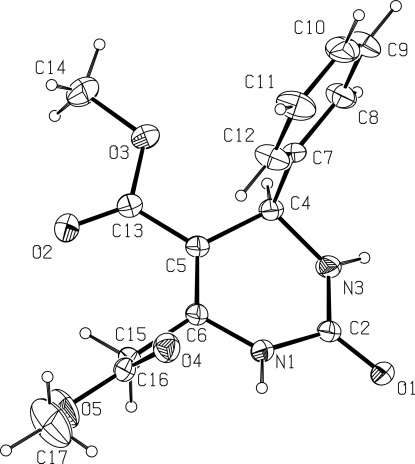
Displacement ellipsoid plot of (1) with the labelling scheme for the non-H atoms, which are drawn as 35% probability ellipsoids.

**Fig. 2.**
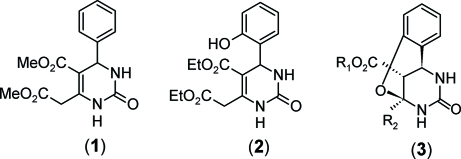
The structures of (1), (2) and (3).

## supplementary crystallographic information

### Comment

Recently, the discovery of monastrol [ethyl 6-methyl-4-(3-hydroxyphenyl)- 2-thioxo-1,2,3,4-tetrahydropyrimidine-5-carboxylate] (Mayer *et al.*, 1999), a lead structure for development of new anticancer agents (Klein *et al.*, 2007), has stimulated considerable interest in preparing its congeners (Haggarty *et al.*, 2000). To follow this research, our strategy was to synthesize conformationally restricted dihydropyrimidine heterocycles by employing a Biginelli-like condensation of salicylaldehyde with dialkyl acetone-1,3-dicarboxylates (Světlík *et al.*, 2008). Unexpectedly, formation of two types of products was detected depending on the ester alkyl group of the starting dialkyl 3-oxopentanedioate: for the methyl ester the reaction proceeded in a 'normal' way to produce the rigid tricyclic structure (3), while the ethyl analogue gave the structure (2), *i.e.* the final cyclization did not occur. To provide structural basis for this product dichotomy, we selected compounds (1) and (2) for a single-crystal X-ray analysis. As the structure of (2) was described previously (Kettmann *et al.*, 2008), we report herein the structure of the title compound (1); it is the de-hydroxy derivative (to prevent cyclization of the molecule) of its normal tricyclic compound.

As mentioned above, the analysis of the structural results (Fig.1, Table 1) is focused on the conformational characteristics of the present structure (1) in comparison with those obtained recently for (2). The comparison has shown that, apart from the conformation of the 5-ester substituent (*cis* and *trans* in (1) and (2), respectively), the only significant difference between (1) and (2) concerns rotation around the ROOC-CH_2_ single bond as measured by the torsion angle C6—C15—C16—O4 which is *ca* 0° in (1) and *ca* 180° in (2). As this is the only difference which affect the vicinity of the critical C6-position, it should be responsible for the dichotomy in the reactivity of methyl and ethyl acetone-1,3-dicarboxylates. Indeed, as shown in Fig.1, the hydroxy oxygen of the 2-hydroxyphenyl derivative of (1) would have an unrestricted access to C6 and hence the oxygen-bridged pyrimidine (3) would easily be formed. By contrast, due to the different geometry of the ester-methylene grouping in (2), the bulky ethoxy moiety points towards the heterocycle where it sterically hinders the nucleophilic addition of the *ortho*-hydroxyl on the pyrimidine C5=C6 double bond, with the net result being the 'open', uncyclized molecule (2).

The crystal packing is governed by hydrogen bonding. As shown in Table 2, there are two independent hydrogen bonds, one joins the molecules into dimers and the other links the dimers into chains.

### Experimental

Synthesis of the title compound, (1), has been described (Světlík *et al.*, 2008). In short, heating of benzaldehyde (0.51 ml, 5 mmol) with dimethyl acetone-1,3-dicarboxylate (0.73 ml, 5 mmol) and urea (0.36 g, 6 mmol) under *p*-toluenesulfonic acid (0.04 g, 0.2 mmol) catalysis without solvent at 353–363 K for 3 h gave desired product (39% yield; m.p. 453–455 K). Crystals suitable for the X-ray analysis were obtained by a slow crystallization from ethanol.

### Refinement

H atoms were visible in difference maps and were subsequently treated as riding atoms with distances C—H = 0.93 Å (CH_arom_), 0.97 (CH_2_) or 0.98 Å (CH), 0.96 Å (CH_3_) and N—H = 0.86 Å; *U*_iso_ of the H atoms were set to 1.2 (1.5 for the methyl H atoms) times *U*_eq_ of the parent atom. Because of the indication from Hirshfeld test (Hirshfeld, 1976), 54 rigid-bond restraints on anisotropic displacement parameters for all bonds involving the non-H atoms were applied during the least-squares refinement. Reflection 110, affected by secondary extinction, was deleted from the refinement.

### Crystal data

**Table:**

C_15_H_16_N_2_O_5_	*F*_000_ = 1280
*M**_r_* = 304.35	*D*_x_ = 1.320 Mg m^−^^3^
Monoclinic, *C*2/*c*	Melting point: 454 K
Hall symbol: -C 2yc	Mo *K*α radiation λ = 0.71073 Å
*a* = 23.498 (5) Å	Cell parameters from 20 reflections
*b* = 12.072 (2) Å	θ = 7–18º
*c* = 10.933 (5) Å	µ = 0.10 mm^−^^1^
β = 99.15 (2)º	*T* = 296 (2) K
*V* = 3061.9 (16) Å^3^	Prism, colourless
*Z* = 8	0.30 × 0.25 × 0.20 mm

### Data collection

**Table:**

Siemens P4 diffractometer	*R*_int_ = 0.040
Radiation source: fine-focus sealed tube	θ_max_ = 30.0º
Monochromator: graphite	θ_min_ = 1.9º
*T* = 296(2) K	*h* = −1→32
ω/2θ scans	*k* = −1→16
Absorption correction: none	*l* = −15→15
5187 measured reflections	3 standard reflections
4466 independent reflections	every 97 reflections
2308 reflections with *I* > 2σ(*I*)	intensity decay: none

### Refinement

**Table:**

Refinement on *F*^2^	Secondary atom site location: difference Fourier map
Least-squares matrix: full	Hydrogen site location: inferred from neighbouring sites
*R*[*F*^2^ > 2σ(*F*^2^)] = 0.046	H-atom parameters constrained
*wR*(*F*^2^) = 0.118	*w* = 1/[σ^2^(*F*_o_^2^) + (0.0575*P*)^2^] where *P* = (*F*_o_^2^ + 2*F*_c_^2^)/3
*S* = 0.90	(Δ/σ)_max_ = 0.001
4466 reflections	Δρ_max_ = 0.13 e Å^−^^3^
201 parameters	Δρ_min_ = −0.27 e Å^−^^3^
54 restraints	Extinction correction: none
Primary atom site location: structure-invariant direct methods	

### Special details

**Table:**

Geometry. All esds (except the esd in the dihedral angle between two l.s. planes) are estimated using the full covariance matrix. The cell esds are taken into account individually in the estimation of esds in distances, angles and torsion angles; correlations between esds in cell parameters are only used when they are defined by crystal symmetry. An approximate (isotropic) treatment of cell esds is used for estimating esds involving l.s. planes.
Refinement. Refinement of *F*^2^ against ALL reflections. The weighted *R*-factor *wR* and goodness of fit *S* are based on *F*^2^, conventional *R*-factors *R* are based on *F*, with *F* set to zero for negative *F*^2^. The threshold expression of *F*^2^ > σ(*F*^2^) is used only for calculating *R*-factors(gt) *etc*. and is not relevant to the choice of reflections for refinement. *R*-factors based on *F*^2^ are statistically about twice as large as those based on *F*, and *R*- factors based on ALL data will be even larger.

### Fractional atomic coordinates and isotropic or equivalent isotropic displacement parameters (Å^2^)

**Table:**

	*x*	*y*	*z*	*U*_iso_*/*U*_eq_	
N1	0.44833 (5)	0.44128 (9)	0.38217 (11)	0.0509 (3)	
H1	0.4615	0.4278	0.4587	0.061*	
C2	0.44596 (5)	0.55052 (11)	0.34279 (13)	0.0477 (3)	
O1	0.47167 (4)	0.62359 (8)	0.40833 (10)	0.0628 (3)	
N3	0.41662 (5)	0.56699 (9)	0.23011 (11)	0.0536 (3)	
H3	0.4214	0.6292	0.1948	0.064*	
C4	0.37661 (5)	0.48718 (11)	0.16121 (13)	0.0479 (3)	
H4	0.3779	0.4979	0.0728	0.058*	
C5	0.39764 (5)	0.37090 (11)	0.19483 (12)	0.0458 (3)	
C6	0.43076 (5)	0.35337 (10)	0.30560 (12)	0.0444 (3)	
C7	0.31513 (5)	0.50992 (12)	0.18200 (12)	0.0473 (3)	
C8	0.28364 (7)	0.59098 (14)	0.11197 (16)	0.0676 (4)	
H8	0.2998	0.6291	0.0520	0.081*	
C9	0.22800 (7)	0.61558 (16)	0.13099 (19)	0.0827 (5)	
H9	0.2069	0.6696	0.0827	0.099*	
C10	0.20409 (7)	0.56203 (17)	0.21879 (18)	0.0825 (5)	
H10	0.1669	0.5795	0.2314	0.099*	
C11	0.23487 (7)	0.48198 (19)	0.28903 (18)	0.0894 (6)	
H11	0.2185	0.4444	0.3491	0.107*	
C12	0.29047 (6)	0.45678 (16)	0.27056 (15)	0.0714 (5)	
H12	0.3113	0.4028	0.3193	0.086*	
C13	0.37881 (6)	0.28141 (13)	0.10727 (13)	0.0535 (3)	
O2	0.39408 (5)	0.18597 (10)	0.11528 (11)	0.0759 (4)	
O3	0.34141 (5)	0.31856 (10)	0.01108 (10)	0.0760 (4)	
C14	0.32059 (10)	0.23753 (17)	−0.08185 (17)	0.1000 (7)	
H14A	0.3526	0.2055	−0.1139	0.150*	
H14B	0.2951	0.2726	−0.1480	0.150*	
H14C	0.3001	0.1805	−0.0457	0.150*	
C15	0.45308 (6)	0.24370 (11)	0.35961 (13)	0.0513 (3)	
H15A	0.4440	0.1867	0.2971	0.062*	
H15B	0.4947	0.2478	0.3805	0.062*	
C16	0.42908 (6)	0.21132 (11)	0.47094 (14)	0.0522 (3)	
O4	0.39511 (5)	0.26165 (9)	0.51997 (10)	0.0646 (3)	
O5	0.45077 (6)	0.11392 (10)	0.51199 (14)	0.0996 (5)	
C17	0.43033 (14)	0.0714 (2)	0.6208 (3)	0.1504 (12)	
H17A	0.4401	0.1224	0.6882	0.226*	
H17B	0.4481	0.0011	0.6430	0.226*	
H17C	0.3892	0.0623	0.6035	0.226*	

### Atomic displacement parameters (Å^2^)

**Table:**

	*U*^11^	*U*^22^	*U*^33^	*U*^12^	*U*^13^	*U*^23^
N1	0.0531 (6)	0.0410 (5)	0.0532 (6)	−0.0021 (5)	−0.0084 (5)	0.0027 (5)
C2	0.0392 (6)	0.0429 (7)	0.0574 (8)	−0.0001 (6)	−0.0032 (6)	0.0048 (6)
O1	0.0607 (6)	0.0434 (5)	0.0749 (7)	−0.0039 (5)	−0.0177 (5)	−0.0015 (5)
N3	0.0486 (6)	0.0447 (6)	0.0619 (7)	−0.0066 (5)	−0.0078 (5)	0.0122 (5)
C4	0.0415 (6)	0.0464 (7)	0.0527 (8)	−0.0022 (5)	−0.0022 (5)	0.0035 (6)
C5	0.0368 (6)	0.0455 (6)	0.0530 (7)	0.0002 (5)	0.0010 (5)	0.0003 (5)
C6	0.0360 (6)	0.0407 (6)	0.0548 (7)	0.0011 (5)	0.0017 (5)	0.0003 (5)
C7	0.0390 (6)	0.0490 (7)	0.0490 (7)	0.0024 (5)	−0.0080 (5)	−0.0046 (6)
C8	0.0557 (8)	0.0689 (11)	0.0743 (10)	0.0115 (7)	−0.0019 (7)	0.0143 (8)
C9	0.0596 (9)	0.0825 (12)	0.0996 (14)	0.0289 (9)	−0.0070 (8)	0.0080 (10)
C10	0.0446 (8)	0.1046 (15)	0.0955 (13)	0.0202 (9)	0.0028 (8)	−0.0154 (10)
C11	0.0569 (9)	0.1245 (17)	0.0893 (13)	0.0169 (10)	0.0190 (9)	0.0189 (11)
C12	0.0502 (8)	0.0883 (12)	0.0746 (11)	0.0138 (8)	0.0064 (7)	0.0244 (9)
C13	0.0462 (7)	0.0525 (8)	0.0594 (8)	−0.0017 (6)	0.0006 (6)	−0.0029 (6)
O2	0.0868 (8)	0.0517 (6)	0.0811 (8)	0.0034 (6)	−0.0118 (6)	−0.0112 (5)
O3	0.0810 (8)	0.0686 (7)	0.0670 (7)	0.0045 (6)	−0.0236 (6)	−0.0132 (5)
C14	0.1211 (17)	0.0911 (14)	0.0731 (11)	−0.0022 (13)	−0.0300 (11)	−0.0234 (10)
C15	0.0430 (7)	0.0420 (7)	0.0655 (8)	0.0065 (6)	−0.0020 (6)	0.0002 (6)
C16	0.0445 (7)	0.0347 (7)	0.0735 (9)	−0.0071 (6)	−0.0023 (6)	0.0033 (6)
O4	0.0629 (6)	0.0556 (6)	0.0764 (7)	−0.0047 (5)	0.0143 (5)	0.0000 (5)
O5	0.1040 (10)	0.0554 (7)	0.1434 (12)	0.0181 (7)	0.0316 (9)	0.0462 (8)
C17	0.172 (3)	0.1040 (19)	0.187 (3)	0.0252 (18)	0.065 (2)	0.0924 (19)

### Geometric parameters (Å, °)

**Table:**

N1—C6	1.3739 (16)	C10—H10	0.9300
N1—C2	1.3856 (17)	C11—C12	1.387 (2)
N1—H1	0.8600	C11—H11	0.9300
C2—O1	1.2324 (16)	C12—H12	0.9300
C2—N3	1.3276 (17)	C13—O2	1.2058 (18)
N3—C4	1.4677 (16)	C13—O3	1.3362 (17)
N3—H3	0.8600	O3—C14	1.4388 (19)
C4—C5	1.5139 (19)	C14—H14A	0.9600
C4—C7	1.5228 (18)	C14—H14B	0.9600
C4—H4	0.9800	C14—H14C	0.9600
C5—C6	1.3480 (19)	C15—C16	1.473 (2)
C5—C13	1.464 (2)	C15—H15A	0.9700
C6—C15	1.5093 (17)	C15—H15B	0.9700
C7—C12	1.365 (2)	C16—O4	1.1957 (17)
C7—C8	1.3824 (19)	C16—O5	1.3308 (17)
C8—C9	1.388 (2)	O5—C17	1.446 (3)
C8—H8	0.9300	C17—H17A	0.9600
C9—C10	1.352 (3)	C17—H17B	0.9600
C9—H9	0.9300	C17—H17C	0.9600
C10—C11	1.368 (3)		
			
C6—N1—C2	123.54 (11)	C10—C11—C12	119.97 (19)
C6—N1—H1	118.2	C10—C11—H11	120.0
C2—N1—H1	118.2	C12—C11—H11	120.0
O1—C2—N3	124.51 (13)	C7—C12—C11	121.07 (16)
O1—C2—N1	120.59 (12)	C7—C12—H12	119.5
N3—C2—N1	114.84 (12)	C11—C12—H12	119.5
C2—N3—C4	125.00 (11)	O2—C13—O3	121.92 (14)
C2—N3—H3	117.5	O2—C13—C5	127.01 (14)
C4—N3—H3	117.5	O3—C13—C5	111.07 (13)
N3—C4—C5	109.05 (10)	C13—O3—C14	115.78 (13)
N3—C4—C7	110.52 (11)	O3—C14—H14A	109.5
C5—C4—C7	114.30 (11)	O3—C14—H14B	109.5
N3—C4—H4	107.6	H14A—C14—H14B	109.5
C5—C4—H4	107.6	O3—C14—H14C	109.5
C7—C4—H4	107.6	H14A—C14—H14C	109.5
C6—C5—C13	122.87 (12)	H14B—C14—H14C	109.5
C6—C5—C4	118.89 (11)	C16—C15—C6	113.64 (12)
C13—C5—C4	118.18 (11)	C16—C15—H15A	108.8
C5—C6—N1	120.05 (12)	C6—C15—H15A	108.8
C5—C6—C15	127.18 (12)	C16—C15—H15B	108.8
N1—C6—C15	112.77 (11)	C6—C15—H15B	108.8
C12—C7—C8	118.39 (14)	H15A—C15—H15B	107.7
C12—C7—C4	122.75 (12)	O4—C16—O5	123.03 (15)
C8—C7—C4	118.80 (13)	O4—C16—C15	127.31 (13)
C7—C8—C9	120.14 (17)	O5—C16—C15	109.66 (14)
C7—C8—H8	119.9	C16—O5—C17	115.58 (17)
C9—C8—H8	119.9	O5—C17—H17A	109.5
C10—C9—C8	120.80 (17)	O5—C17—H17B	109.5
C10—C9—H9	119.6	H17A—C17—H17B	109.5
C8—C9—H9	119.6	O5—C17—H17C	109.5
C9—C10—C11	119.62 (17)	H17A—C17—H17C	109.5
C9—C10—H10	120.2	H17B—C17—H17C	109.5
C11—C10—H10	120.2		
			
C6—N1—C2—O1	−166.61 (13)	C12—C7—C8—C9	−1.0 (2)
C6—N1—C2—N3	10.7 (2)	C4—C7—C8—C9	−178.35 (15)
O1—C2—N3—C4	−167.13 (13)	C7—C8—C9—C10	0.8 (3)
N1—C2—N3—C4	15.7 (2)	C8—C9—C10—C11	−0.6 (3)
C2—N3—C4—C5	−32.53 (18)	C9—C10—C11—C12	0.5 (3)
C2—N3—C4—C7	93.93 (15)	C8—C7—C12—C11	1.0 (3)
N3—C4—C5—C6	25.51 (17)	C4—C7—C12—C11	178.18 (16)
C7—C4—C5—C6	−98.74 (14)	C10—C11—C12—C7	−0.7 (3)
N3—C4—C5—C13	−157.16 (12)	C6—C5—C13—O2	−7.8 (2)
C7—C4—C5—C13	78.58 (16)	C4—C5—C13—O2	174.99 (14)
C13—C5—C6—N1	178.17 (13)	C6—C5—C13—O3	173.11 (13)
C4—C5—C6—N1	−4.6 (2)	C4—C5—C13—O3	−4.10 (18)
C13—C5—C6—C15	−1.0 (2)	O2—C13—O3—C14	0.1 (2)
C4—C5—C6—C15	176.17 (12)	C5—C13—O3—C14	179.23 (16)
C2—N1—C6—C5	−15.8 (2)	C5—C6—C15—C16	−114.21 (16)
C2—N1—C6—C15	163.47 (12)	N1—C6—C15—C16	66.54 (15)
N3—C4—C7—C12	−94.68 (17)	C6—C15—C16—O4	0.0 (2)
C5—C4—C7—C12	28.79 (19)	C6—C15—C16—O5	179.74 (12)
N3—C4—C7—C8	82.53 (15)	O4—C16—O5—C17	0.3 (3)
C5—C4—C7—C8	−154.00 (13)	C15—C16—O5—C17	−179.39 (18)

### Hydrogen-bond geometry (Å, °)

**Table:**

*D*—H···*A*	*D*—H	H···*A*	*D*···*A*	*D*—H···*A*
N1—H1···O1^i^	0.86	2.06	2.8326 (17)	149
N3—H3···O4^ii^	0.86	2.32	3.0730 (17)	146

Symmetry codes: (i) −*x*+1, −*y*+1, −*z*+1; (ii) *x*, −*y*+1, *z*−1/2.

## References

[bb1] Haggarty, S. J., Mayer, T. U., Miyamoto, D. T., Fathi, R., King, R. W., Mitchison, T. J. & Schreiber, S. L. (2000). *Chem. Biol.* **7**, 275–286. 10.1016/s1074-5521(00)00101-0 10780927

[bb2] Hirshfeld, F. L. (1976). *Acta Cryst.* A**32**, 239–244.

[bb3] Kettmann, V., Světlík, J. & Veizerová, L. (2008). *Acta Cryst.* E**64**, o1092. 10.1107/S1600536808012683 PMC2961528 21202607

[bb4] Klein, E., DeBonis, S., Thiede, B., Skoufias, D. A., Kozielski, F. & Lebeau, L. (2007). *Bioorg. Med. Chem.* **15**, 6474–6488. 10.1016/j.bmc.2007.06.016 17587586

[bb5] Mayer, T. U., Kapoor, T. M., Haggarty, S. J., King, R. W., King, R. W., Schreiber, S. L. & Mitchison, T. J. (1999). *Science*, **286**, 971–974. 10.1126/science.286.5441.971 10542155

[bb6] Sheldrick, G. M. (2008). *Acta Cryst.* A**64**, 112–122. 10.1107/S0108767307043930 18156677

[bb7] Siemens (1991). *XSCANS* Siemens Analytical X-ray Instruments Inc., Madison, Wisconsin, USA.

[bb8] Spek, A. L. (2003). *J. Appl. Cryst.* **36**, 7–13.

[bb9] Světlík, J., Veizerová, L. & Kettmann, V. (2008). *Tetrahedron Lett.* **49**, 3520–3523.

